# The Induction of Renal Tumours by Feeding Lead Acetate to Rats

**DOI:** 10.1038/bjc.1962.33

**Published:** 1962-06

**Authors:** E. Boyland, C. E. Dukes, P. L. Grover, B. C. V. Mitchley

## Abstract

**Images:**


### THE INDUCTION OF RENAL TUMOURS BY FEEDING LEAD ACETATE TO RATS

E. BOYLAND, C. E. DUKES, P. L. GROVER AND B. C. V. MITCHLEY

*From the Chester Beatty Research Institute, Institute of Cancer Research : Royal Cancer Hospital, Fulham Road, London, S.W.3*

Received for publication December 7, 1961

IN a careful examination of the long-term effects of large doses of lead, Zollinger (1953) described the tumours of the kidney in rats induced by repeated injection of lead phosphate. This finding has been confirmed by Walpole (personal communication in Matthews and Walpole, 1958) who also obtained tumours in the kidney by injection of lead phosphate. The tumours were similar to those caused by administration of 4-amino-5-fluorobiphenyl (Matthews and Walpole, 1958).

Van Esch, van Genderen and Vink (1959, personal communication) fed rats on a diet containing 1 per cent of basic lead acetate over a period of two years. At the end of this period the surviving rats were killed and many were found to have malignant tumours of the kidney. That rats should survive for two years on a diet containing 1 per cent lead acetate is surprising, in view of the known toxicity of lead compounds. In this paper, the findings of van Esch, van Genderen and Vink (1959, personal communication) are confirmed.

Fairhall and Miller (1941) had previously carried out experiments with much lower concentrations of lead. They maintained rats on diets containing 0·1 per cent of lead arsenate and 0·1 per cent lead carbonate for two years. The mortality in the group fed the lead carbonate was almost the same as in the controls. At the end of the two year period, the kidneys of the rats were found to have many swollen cells with large vesicular nuclei and brown granules ; the latter were most prominent in the proximal convoluted tubules, but no neoplasms were reported. The concentration of lead in the kidney was higher than in the liver but lower than in bone.

Lead compounds are known to cause increased excretion of porphyrins by interfering with haemoglobin metabolism. As the porphyrins pass through the kidney they might be the immediate carcinogen, rather than the administered lead. Lead is deposited in bones, so that if the lead itself were carcinogenic then bone tumours would be expected. The hypothesis that porphyrins might be the immediate carcinogens in the kidney was tested by administering allylisopropylacetylcarbamide (Sedormid), which also causes porphyrinuria, to rats. The porphyrin concentration in the urine of rats dosed with lead acetate, sedormid and other substances known to cause porphyrinuria was measured.

*Experimental*

Two groups of 20 male 10-week-old Wistar rats were maintained on a 20 per cent protein diet of the following composition ; white flour, 68·7 per cent ; casein, 11·5 per cent ; milk powder, 8 per cent ; margarine, 3·3 per cent ; chalk, 1·3 per cent ; salt mixture (Glaxo Laboratories), 0·8 per cent ; " Bemax ", 2·5 per cent ; cod liver oil, 1·5 per cent ; dried yeast, 2·4 per cent. Lead acetate (1 per cent w/w) was mixed with the dry diet for one group and sedormid (0·5 per cent w/w) with that for the other group. The diets were mixed to a dough with water before feeding and drinking water was supplied *ad libitum*. The lead acetate and sedormid containing diets were fed at the rate of 20 g. of dry diet/rat/day for the first month, 30 g./rat/day for the second month and then 40 g./rat/day for the succeeding 10 months. After 1 year the lead and sedormid diets were replaced by the basic 20 per cent protein diet. The rats were individually weighed at regular intervals and on both the lead and sedormid diets showed a steady increase in weight from 200 g. at the start of the experiment to 600–800 g. after 1 year. The animals were palpated regularly and were killed when they appeared to be ill with marked loss of weight, or when a tumour was palpable.

TABLE I.—*Lead Acetate Feeding Experiment*

Twenty male rats fed a 20 per cent protein diet containing 1 per cent lead acetate for up to one year

| Survival time in days | Pathology |
|---|---|
| 77 | Hydronephrosis. |
| 105 | Pyelonephritis. |
| 126 | Decomposed. |
| 146 | Nothing abnormal detected. |
| 331 | Right kidney—renal carcinoma. |
| 335 | Bilateral renal tumours with adrenal involvement. |
| 335 | Bilateral cuboidal cell carcinoma of kidney. |
| 335 | Bilateral cuboidal cell carcinoma of kidney. |
| 350 | Nothing abnormal detected. |
| 358 | Bilateral cuboidal cell carcinoma of kidney. |
| 368 | Right kidney cuboidal cell carcinoma. |
| 436 | Papillary carcinoma of right kidney. |
| 456 | Bilateral renal cuboidal cell carcinoma. |
| 476 | Renal cuboidal cell carcinoma ?, bilateral. |
| 484 | Bilateral renal cuboidal cell carcinoma. |
| 519 | Bilateral renal carcinoma (cuboidal cell). |
| 543 | Bilateral renal cuboidal cell carcinoma. |
| 582 | Papillary carcinoma—bilateral. |
| 606 | Renal carcinoma—bilateral : histologically variable. |
| 629 | Bilateral renal carcinoma. |

### EXPLANATION OF PLATES

FIG. 1.—" Cystic nephritis " in rat fed for one year on sedormid diet. Death 18 months after commencement of experiment. No tumours.

FIG. 2.—Early focus of carcinoma (marked by arrow) in kidney of rat fed for 11 months on lead acetate diet. " Cystic nephritis " also present.

FIG. 3.—Large focus of carcinoma in kidney of rat fed for one year on lead acetate diet. Severe " cystic nephritis " also present. Rat died 19 months after commencement of experiment.

FIG. 4.—Two large carcinomas in kidney of rat fed for one year on lead acetate diet. Rat died 15 months after commencement of experiment.

FIG. 5.—Margin of solid cuboidal cell renal carcinoma in rat fed for one year on lead acetate diet. $\times 90$.

FIG. 6.—Higher magnification of tumour illustrated in Fig. 5. $\times 590$.

FIG. 7.—Tubular pattern developing in cuboidal cell renal carcinoma. Rat died 14 months after commencement of lead acetate diet. $\times 90$.

FIG. 8.—Papillary pattern at margin of large renal carcinoma in rat 15 months after commence of experiment. $\times 90$.

1 2 3 4

Boyland Dukes, Grover and Mitchley.

5

6

7

8

TABLE II.—*Sedormid Feeding Experiment*

Twenty male rats fed a 20 per cent protein diet containing 0·5 per cent sedormid for up to one year

| Survival time in days | Pathology |
|---|---|
| 104 | Pyelonephritis. |
| 137 | Nothing abnormal detected. |
| 165 | Interstitial nephritis with toxic liver. |
| 345 | Hyaline casts—kidney. Fatty liver. |
| 345 | Killed, worm infection. No neoplasm. |
| 363 | Abscess in lung. Fatty liver. Cystic dilatation of tubules. |
| 373 | Bilateral hydronephrosis and pyelonephritis. |
| 464 | Nothing abnormal detected. |
| 488 | Invasive carcinoma of kidney—small foci. |
| 506 | Cystic nephritis. |
| 506 | ,, ,, |
| 534 | Nothing abnormal detected. |
| 569 | Nephritis. |
| 595 | Chronic nephritis. |
| 596 | ,, ,, |
| 616 | ,, ,, |
| 679 | ,, ,, |
| 692 | Nephritis. |
| 702 | Hydronephritis. |
| 702 | Nephritis. |

TABLE III.—*Excretion of Urinary Coproporphyrin III by Male Rats*

| Treatment | Coproporphyrin ($\mu$g./2 rats/day) Young rats (200 g.) | Adult rats (650 g.) |
|---|---|---|
| Untreated controls | 5·6 | 12·6 |
| Lead acetate (1% of diet) | — | 93 |
| 16 days after cessation of lead diet | — | 33 |
| Sedormid (0·5% of diet) | — | 233 |
| 3 days after cessation of sedormid diet | — | 30 |
| 1,4-Dihydro-2,4,6-trimethyl-3,5-dicarbethoxypyridine | | |
| (*a*) 0·5% of diet | 14·6 | — |
| (*b*) 1·0% of diet | 20·0 | — |
| Ethyl methane sulphonate (200 mg./kg.) by subcutaneous injection (mean for 3 days after single injection) | 86·6 | — |
| 4-Fluoro-4′-aminodiphenyl (100 mg./kg.) by subcutaneous injection (mean for 3 days after a single injection) | 8·4 | — |

*Porphyrin excretion*

Urine was collected from pairs of male rats housed in all-glass metabolism cages, and the urinary coproporphyrin III determined spectrophotometrically (Rimington, 1958). The results of these determinations are given in Table III.

*Description of renal lesions caused by lead acetate and sedormid*

Both lead acetate and sedormid given orally to rats caused chronic cystic nephritis ; in the lead acetate animals this was followed by neoplasms, whereas in the sedormid animals it was not. In the lead acetate series 16 out of 20 animals lived for 320 days or more and 15 of these ultimately developed kidney tumours, either adenomas or carcinomas ; the tumours were often multiple and bilateral.

In the sedormid series only one neoplastic renal lesion was found, but this was an epidermoid carcinoma of the renal pelvis at an early stage of development and not a tumour of the renal parenchyma. Another animal had an interstitial cell tumour of the testicle, but both these lesions were probably incidental findings and not related to the experiment.

After a period of about 6 months all the rats in the experiments, whether given lead acetate or sedormid, developed a lesion which we have called chronic cystic nephritis. The kidneys were slightly enlarged, appeared granular and on section were found to contain innumerable small cysts (Fig. 1). These were lined with cuboidal or flattened epithelium and often contained hyaline eosinophilic material. The lesion was obviously due to a cystic dilatation of the renal tubules accompanied by a slight degree of interstitial fibrosis. The glomeruli and blood vessels did not appear to be affected.

The animals were kept alive as long as possible to see how many eventually developed tumours, so that the initial stages of these renal lesions were not seen, but early effects on the kidney of ingested lead have been previously recorded. Finner and Calvery (1939) made a series of pathological examinations on groups of rats receiving diets containing different lead compounds, including lead acetate. They found that the kidneys showed marked irregularity of the tubular epithelium with hypertrophy of the nuclei, some of which contained eosinophilic inclusion bodies similar to those known to occur in the kidneys in cases of lead poisoning in man. Pardoe (1952) found that during the first three months of treatment with lead, microscopic examination revealed little change in the kidneys of rats, but after 4 to 5 months degenerative changes were evident in the renal tubules, particularly in the deep cortex towards the boundary zone. The second convoluted tubules were also affected and often dilated and lined by flattened regenerating epithelium. The blood vessels and glomeruli appeared normal and no changes were found in the interstitial tissue apart from focal infiltration with small lymphocytes in relation to the most damaged tubules.

In the present experiment, 4 of the 20 rats fed on the lead acetate diet died within 6 months and showed degenerative changes in the kidneys but no neoplasms. The first renal tumour was found in a rat which had been fed the lead acetate diet for 11 months. The animal was killed because it was obviously ill, and at post-mortem examination a small solid nodule was noticed in the right kidney. On section this was found to be an early focus of cuboidal cell carcinoma (Fig. 2).

Four more rats which had received the lead acetate diet for 11 months were killed and dissected within the next few days and in 3, renal tumours were found.

Eleven of the 20 rats survived for more than 12 months. These were killed when a tumour could be palpated or when the animal appeared to be ill, and renal carcinoma was found in each of these eleven animals. Two of these are illustrated in Fig. 3 and Fig. 6. The tumours were often bilateral and associated with small adenomas, hyperplastic foci and nodules of regenerating tubular epithelium. The smaller neoplastic lesions were usually solid collections of cuboidal cells (Fig. 5 and Fig. 6) but the larger tumours tended to develop a tubular (Fig. 7) or papillary pattern (Fig. 8) with vacuolated cells similar to those of human renal carcinomas.

### DISCUSSION

The experiments extend the findings of Zollinger (1953) and Matthews and Walpole (1958), and confirm those of van Esch, van Genderen and Vink (1959, personal communication) that ingested lead induces cancer of the kidney in rats. The significance of the results in relation to other causes of renal tumours has been discussed elsewhere (Dukes, 1961). They leave open the question whether the actual carcinogen is a lead derivative or porphyrin (or possibly a lead porphyrin). The failure of sedormid to induce kidney tumours might be due to different types of porphyria being caused by lead salts, and sedormid. In another investigation Connell (1961, personal communication) treated mice with ethyl methanesulphonate and obtained a number of kidney tumours. The injection of ethyl methanesulphonate into rats caused marked porphyrinuria (Table III) so that in this case the actual carcinogen could be a porphyrin. Treatment with 4'-amino-4-fluorobiphenyl did not increase porphyrin excretion, but with this compound the actual carcinogen is probably an excreted metabolite—either an aminophenol or aryl hydroxylamine derivative.

Although lead salts and sedormid both induce porphyrinuria the modes of action are probably different. Sedormid appears to inhibit the conversion of porphyrins into catalase and haemoglobin (Schmid and Schwarz, 1956) so that the synthesised porphyrins are not utilised but excreted. On the other hand, lead appears to cause breakdown of haemoglobin and increase in porphyrins in this way (cf. Goldblatt, 1955). The immediate carcinogen might be a lead porphyrin. Other substances which induce porphyria are being examined for carcinogenic activity.

Although the carcinogenic activity of lead phosphate and lead acetate have been clearly demonstrated, tumours have only been induced with large doses of the compounds. Tests should be carried out with lead salts at lower levels and with derivatives of other metals, particularly tin, antimony and zinc. Investigations which might provide evidence that exposure to lead presents an occupational cancer hazard for man have not been made, but mortality from renal cancer has been increasing in males during th past decades in England and Wales (Case, 1956). The increase could be due to some environmental factor.

Because the repeated demonstration that the administration of lead salts induces renal cancer in rats, the possiblity that lead derivatives have caused cancer in man should be examined. The fact that the same carcinogen can induce cancer at different sites in different species (e.g. benzidine causes bladder cancer in man and liver cancer in rats) indicates that tumours of all sites should be looked for in men who have been exposed to lead. As lead derivatives damage bone marrow they might induce leukaemia and it would be important to look for blood abnormalities as well as tumours of all sites in an epidemiological study.

The amount of lead in road dust and in the air of towns has increased greatly since lead tetraethyl has been added to petrol. Thus, the lead content of the air of Zurich has increased from 1·4 $\mu$g. per m$^3$ in 1949–50 (before lead tetraethyl was used) to 4·5 $\mu$g. per m$^3$ in 1960–61 (Eidg. Bleibenzin Kommission, 1961). People living in cities absorb 20–30 $\mu$g. of lead per day from their inspired air. This is small compared with the 200–300 $\mu$g. per day of lead which is the usual intake of lead from food for Europeans.

SUMMARY

1. Twenty male rats fed on a diet containing 1 per cent lead acetate for one year excreted porphyrin and developed cystic nephritis. Of 16 rats which survived 320 days, 15 were found to have either adenomas or adenocarcinomas of the kidney.

2. Twenty male rats fed on a diet containing sedormid for one year excreted large quantities of porphyrin and also developed cystic nephritis. No tumours of the kidney parenchyma occurred in them but an early form of transitional cell carcinoma of the renal pelvis was noticed in one animal.

We are indebted to Dr. J. Marks of Roche Products Ltd., Welwyn Garden City, for a gift of sedormid used in the experiments. This investigation has been supported by grants to the Chester Beatty Research Institute (Institute of Cancer Research : Royal Cancer Hospital) from the Medical Research Council, the British Empire Cancer Campaign, the Anna Fuller Fund, and the National Cancer Institute of the National Institutes of Health, U.S. Public Health Service.

### REFERENCES

CASE, R. A. M.—(1956) *Brit. J. prev. soc. Med.*, **10**, 172.
DUKES, C. E.—(1961) *Lancet*, ii, 1157.
EIDG. BLEIBENZIN KOMMISSION—(1961) Mitteilungen as dem Gebiete Lebensmittel Untersuchung und Hygiene, Heft 3.
FAIRHALL, L. T. AND MILLER, J. W.—(1941) *Publ. Hlth Rep., Wash.*, **56**, 1610.
FINNER, L L. AND CALVERLY, H. O.—(1939) *Arch. Path.*, **27**, 433.
GOLDBLATT, M. W.—(1955) *Brit. J. industr. Med.*, **12**, 1.
MATTHEWS, J. J. AND WALPOLE, A. C.—(1958) *Brit. J. Cancer*, **12**, 234.
PARDOE, A. U.—(1952) *Brit. J. Pharmacol.*, **7**, 349.
RIMINGTON, C.—(1958) Broadsheet 21, The Association of Clinical Pathologists.
SCHMID, R. AND SCHWARZ, S.—(1956) Ciba Symposium ' Porphyrin biosynthesis and Metabolism', London (Churchill), p. 196.
ZOLLINGER, H. V.—(1953) *Virchows Arch.*, **323**, 694.


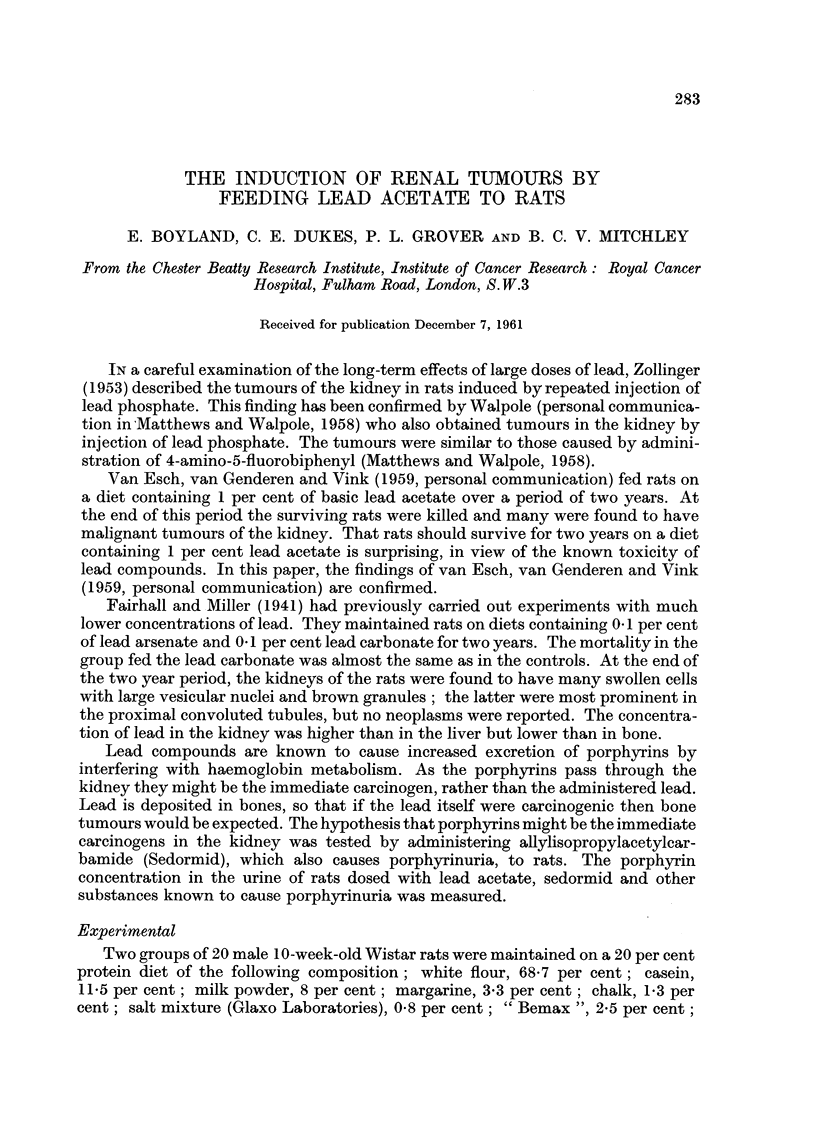

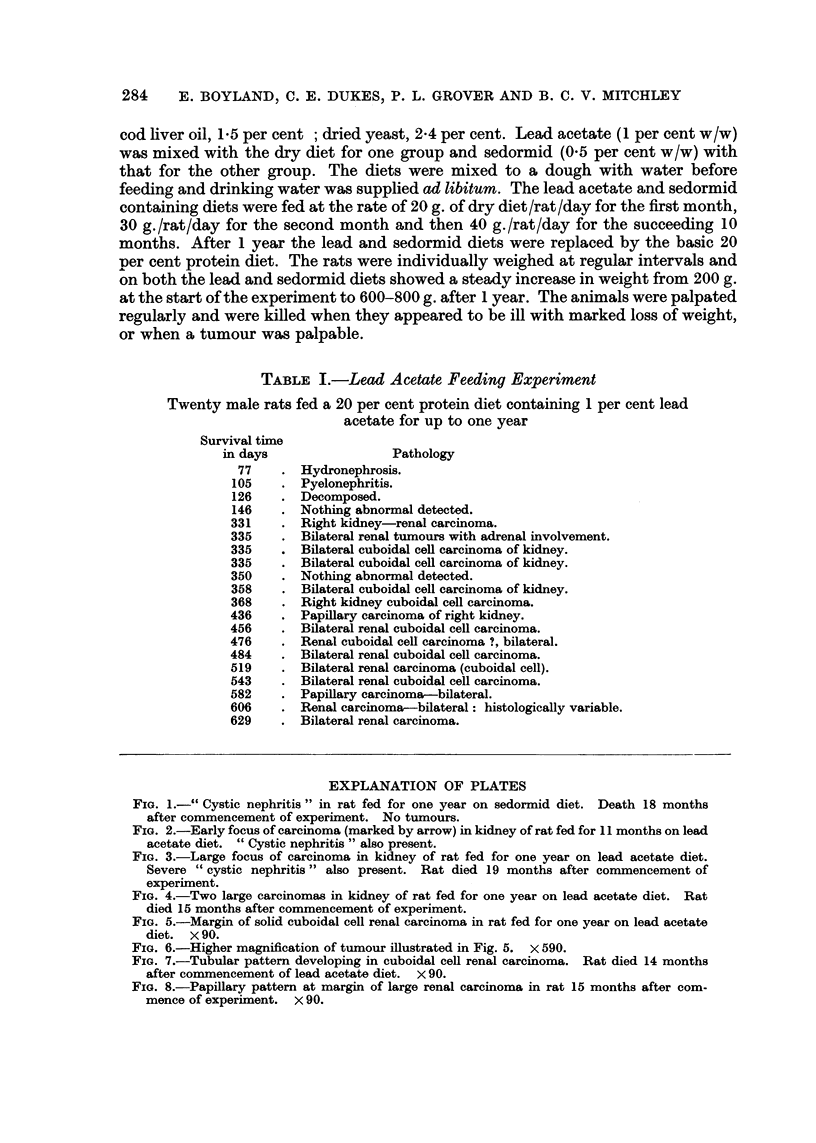

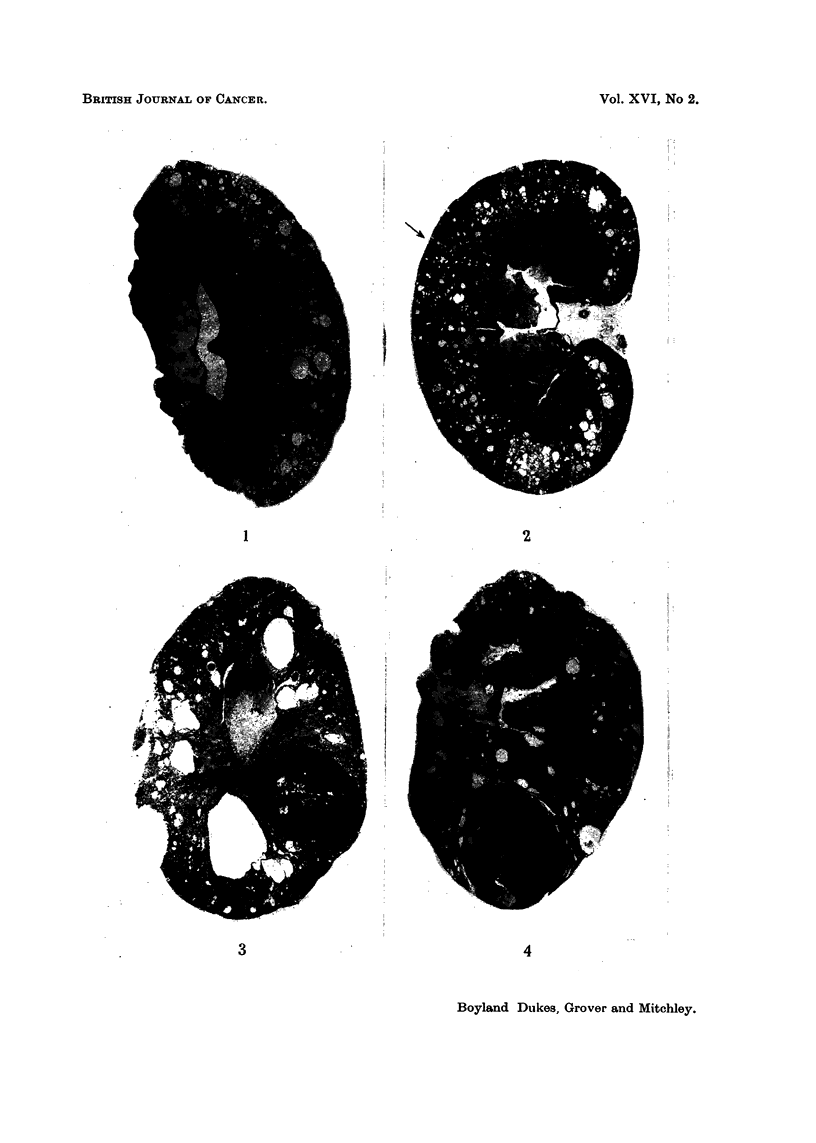

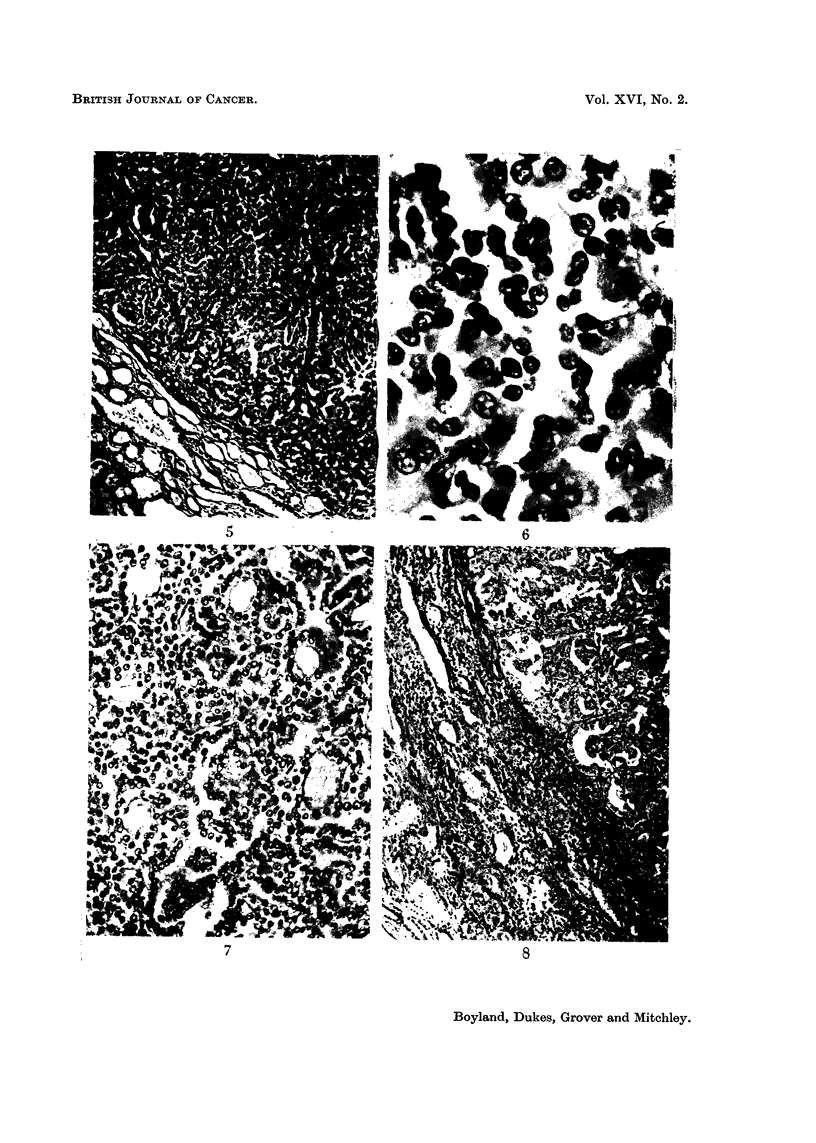

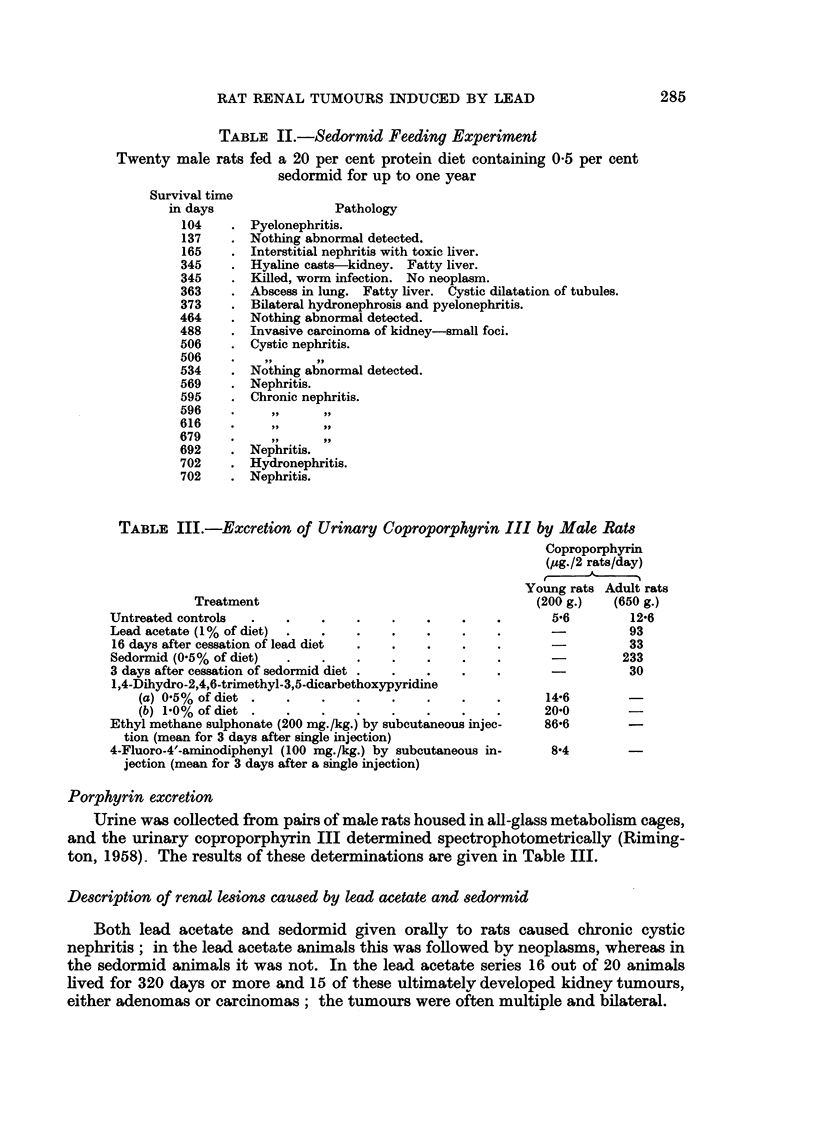

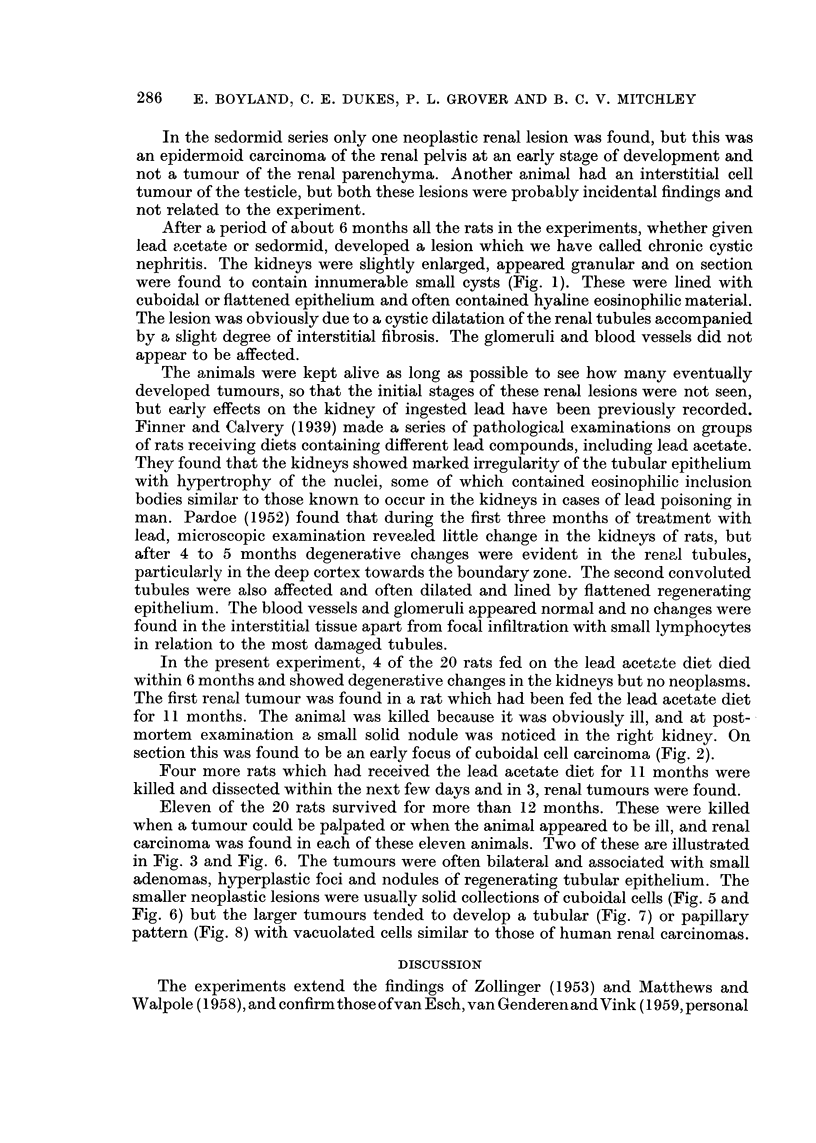

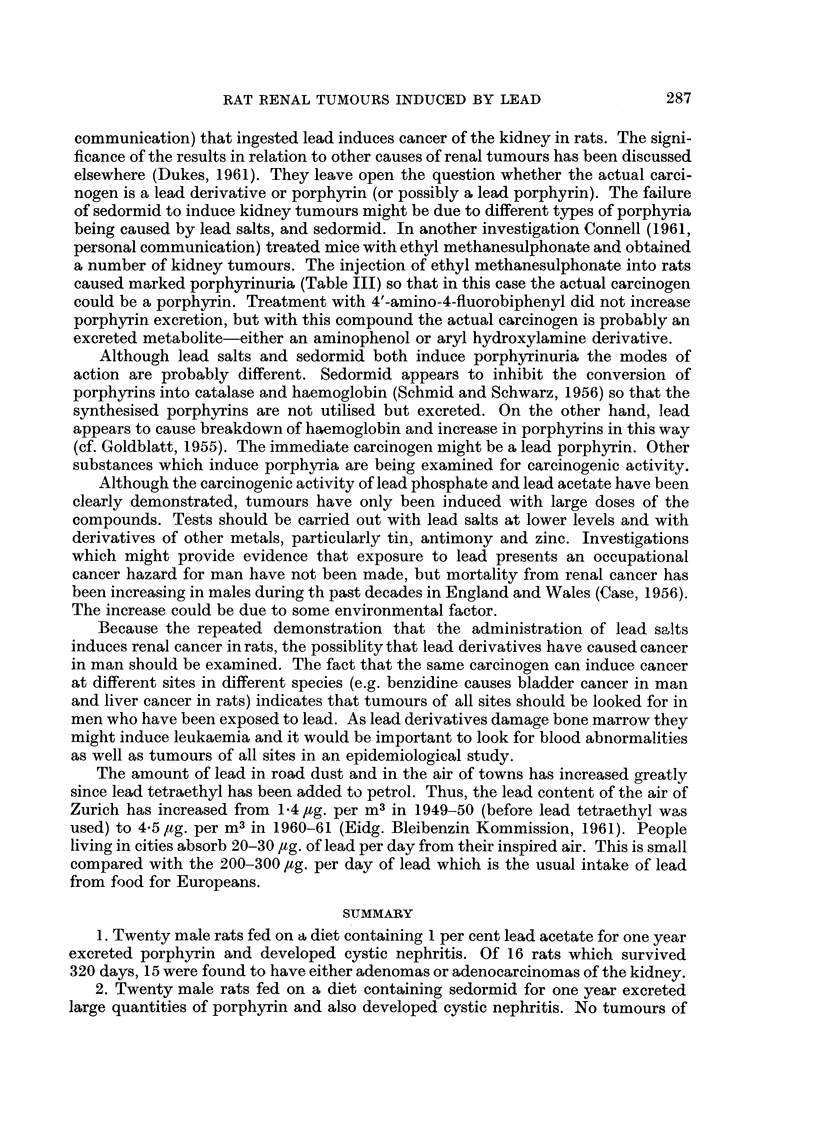

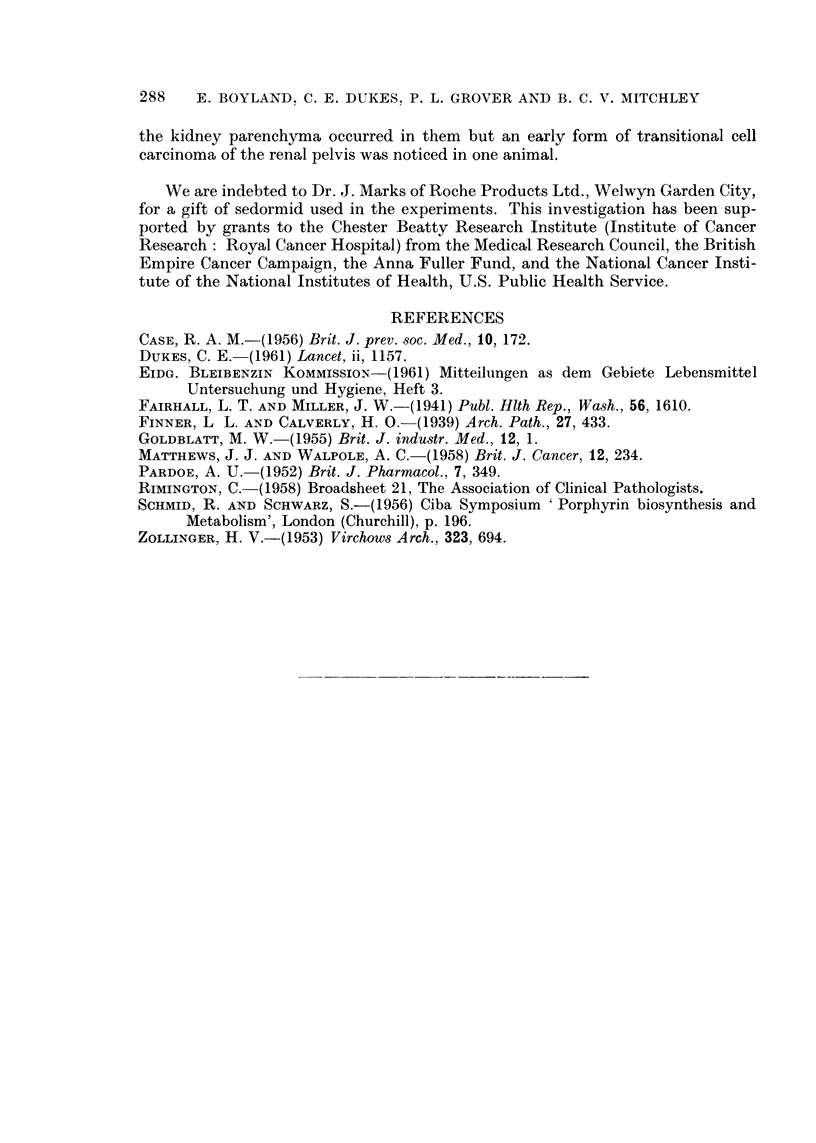


## References

[OCR_00448] CASE R. A. (1956). Cohort analysis of cancer mortality in England and Wales; 1911-1954 by site and sex.. Br J Prev Soc Med.

[OCR_00449] DUKES C. E. (1961). Clues to the causes of cancer of the kidney.. Lancet.

[OCR_00459] MATTHEWS J. J., WALPOLE A. L. (1958). Tumours of the liver and kidney induced in Wistar rats with 4'-fluoro-4-aminodiphenyl.. Br J Cancer.

[OCR_00460] PARDOE A. U. (1952). Renal function in lead poisoning.. Br J Pharmacol Chemother.

[OCR_00468] ZOLLINGER H. U. (1953). Durch chronische Bleivergiftung erzeugte Nierenadenome und -carcinome bei Ratten und ihre Beziehungen zu den entsprechenden Neubildungen des Menschen.. Virchows Arch Pathol Anat Physiol Klin Med.

